# Modulation of *rosR* Expression and Exopolysaccharide Production in *Rhizobium leguminosarum* bv. *trifolii* by Phosphate and Clover Root Exudates

**DOI:** 10.3390/ijms12064132

**Published:** 2011-06-22

**Authors:** Monika Janczarek, Anna Skorupska

**Affiliations:** Department of Genetics and Microbiology, Institute of Microbiology and Biotechnology, University of Maria Curie-Skłodowska, Akademicka 19 st., Lublin, 20–033, Poland; E-Mail: anna.skorupska@poczta.umcs.lublin.pl

**Keywords:** rosR expression, exopolysaccharide synthesis, phosphate, root exudates, *Rhizobium leguminosarum* bv. *trifolii*

## Abstract

The acidic exopolysaccharide (EPS) secreted in large amounts by the symbiotic nitrogen-fixing bacterium *Rhizobium leguminosarum* bv. *trifolii* is required for the establishment of an effective symbiosis with the host plant *Trifolium* spp. EPS biosynthesis in rhizobia is a very complex process regulated at both transcriptional and post-transcriptional levels and influenced by various nutritional and environmental conditions. The *R. leguminosarum* bv. *trifolii rosR* gene encodes a transcriptional regulator with a C_2_H_2_ type zinc-finger motif involved in positive regulation of EPS synthesis. *In silico* sequence analysis of the 450-bp long *rosR* upstream region revealed the presence of several inverted repeats (IR1 to IR6) and motifs with significant identity to consensus sequences recognized by PhoB and LysR-type proteins associated with phosphate- and flavonoid-dependent gene regulation in *R. leguminosarum.* Using a set of sequentially truncated *rosR-lacZ* transcriptional fusions, the role of the individual motifs and the effect of phosphate and clover root exudates on *rosR* expression were established. In addition, the significance of IR4 inverted repeats in the repression, and P2–10 hexamer in the activation of *rosR* transcription, respectively, was found. The expression of *rosR* increased in the presence of phosphate (0.1–20 mM) and clover root exudates (10 μM). PHO boxes and the LysR motif located upstream of the *rosR* translation start site were engaged in the regulation of *rosR* transcription. The synthesis of EPS and biofilm formation decreased at high phosphate concentrations, but increased in the presence of clover root exudates, indicating a complex regulation of these processes.

## 1. Introduction

Exopolysaccharide (EPS) production is a wide spread feature among bacteria, and several functions are ascribed to this polymer, including protection against environmental stress and host defense responses, nutrient gathering, attachment to surfaces, and biofilm formation [1,2]. In nitrogen-fixing symbiotic bacteria, commonly referred to as rhizobia, EPSs are required for successful nodulation of legumes, such as *Trifolium*, *Pisum*, *Vicia* and *Medicago* spp., which form indeterminate-type nodules [3–5]. EPS-deficient mutants of *Rhizobium leguminosarum* and *Sinorhizobium (Ensifer) meliloti* are impaired in the invasion of nodule cells and nitrogen fixation [6–9]. Acidic EPSs secreted in large amounts by rhizobia are species-specific heteropolymers consisting of common sugars substituted with non-carbohydrate residues [4,5,10,11]. Biosynthesis of *R. leguminosarum* EPS is a multi-step process requiring the activity of several enzymes, most of them encoded by *pss* genes located in the large chromosomal EPS cluster I [12,13]. Similarly to other bacterial EPSs, the EPS of *R. leguminosarum* plays a significant role in biofilm formation, being the major component of the matrix [1,14,15]. Also, the level of EPS polymerization is very important for normal biofilm formation, because *prsD*, *plyB*, and *plyBplyA* mutants, forming significantly longer molecules of this polymer than the wild type, are impaired in biofilm formation [16]. Similarly in *S. meliloti*, galactoglucan (EPS II) (especially its low-molecular-weight fraction) plays a crucial role in biofilm formation [17].

The regulation of EPS biosynthesis in rhizobia is controlled at both transcriptional and post-transcriptional levels and influenced by various nutrient sources and stress conditions. In *S. meliloti*, which produces two types of EPSs, several regulators of succinoglycan (EPS I) and galactoglucan (EPS II) synthesis have been identified on the chromosome (*exoR*, *exoS*, *expR*, *syrM*, *mucR*, and *exoD*) and megaplasmid pSymB (*exsB*, *expG*, and *exoX*) [3,5]. The majority of these regulators are engaged in negative regulation of EPS production; *exoR*, *exoS*, *exsB*, and *exoX* genes negatively influence EPS I synthesis, and *expR* and *mucR* negatively regulate EPS II synthesis [18–20]. ExpG (WggR) is a positive regulator of EPS II [21]. MucR plays a key role in the regulation of both types of EPSs in *S. meliloti* as a positive regulator of EPS I and a negative regulator of EPS II synthesis [20,22]. The biosynthesis of EPS I is stimulated by hyperosmotic stress, very high phosphate concentrations (>150 mM), and limitation of some nutrient sources such as nitrogen and sulfur [23–25]. On the other hand, phosphate limitation stimulates EPS II production [21,25,26], indicating that phosphate concentration is a signal affecting the type of EPS produced by *S. meliloti* [20]. Phosphate starvation stimulates the expression of *exp* genes involved in EPS II synthesis by the action of the phosphate-dependent WggR and PhoB regulatory proteins [20,21,27]. PhoB has been found to regulate the transcription of target genes by binding to PHO boxes, which are identified in the promoters of operons involved in EPS II synthesis [28]. The PHO box consensus has recently been found in a large-scale analysis of Pho-dependent promoters in *S. meliloti* and several other species of proteobacteria [28]. Regulation of EPS II production is further influenced by the Sin quorum sensing system in the presence of the ExpR regulator [29].

Under nitrogen limitation, two proteins, SyrM and NtrC, act as positive regulators of EPS I production in *S. meliloti*. SyrM increases the expression of some *exo* genes involved in the synthesis of this polymer via positive stimulation of *syrA* expression [30,31].

In contrast to *S. meliloti*, the regulatory mechanisms and external factors influencing EPS biosynthesis in *R. leguminosarum* have not been extensively studied. Two genes, *psiA* and *psr*, identified on the symbiotic plasmid pSym of *R. leguminosarum* bv. *phaseoli*, are involved in the regulation of EPS synthesis [32,33]. Multiple *psiA* copies inhibit EPS synthesis, but this effect is overcome in the presence of additional *psr* copies. EPS production is also negatively regulated by *exoR*, which shows extensive similarity to *S. meliloti exoR* [34]. In *R. leguminosarum* bv. *trifolii*, a negative regulatory effect of *pssB*, encoding inositol monophosphate phosphatase, on EPS synthesis has been described [35].

Recently, *rosR* encoding a positive transcriptional regulator of EPS synthesis has been identified in *R. leguminosarum* bv. *trifolii* [36]. It shares significant identity with *mucR* of *S. meliloti* [37], *rosR* of *Rhizobium etli* [38], *rosAR* of *Agrobacterium radiobacter* [39], and *ros* of *Agrobacterium tumefaciens* [40]. All these genes encode transcriptional regulators belonging to the family of Ros/MucR proteins, which have the Cys_2_His_2_ type zinc-finger motif and are required for positive or negative regulation of EPS biosynthesis. Mutation in *R. leguminosarum* bv. *trifolii rosR* results in substantially diminished amounts of EPS and ineffective symbiosis with clover, whereas multiple copies of this gene cause a nearly two-fold increase of EPS production [36,41]. RosR is a 15.7 kDa protein, which by binding to the 22-bp-long RosR-box sequence located in the *rosR* upstream region decreases *rosR* transcription, demonstrating autoregulation of its own gene [36]. Besides RosR-box, two P1 and P2 promoters, and three motifs with significant identity to *E. coli* consensus sequence binding the cAMP-CRP complex have been found in the promoter region [42]. Also, the significance of an AT-rich UP element located upstream of −35 hexamer and extended −10 TGG motif in an enhancement of the *rosR* transcription initiation from the P1 promoter was confirmed [42]. In the presence of glucose, *rosR* transcription has been significantly decreased showing the possibility of regulation of *rosR* expression by catabolic repression. It has been established that mutation in *rosR* resulted in several pleiotropic phenotypes, such as quantitative alterations in the polysaccharide constituent of lipopolysaccharide, changes in membrane and secreted protein profiles, and higher sensitivity to surface-active detergents and some osmolytes [43]. In addition, the *rosR* mutant has been shown to exhibit dramatically decreased attachment to and colonization of clover root hairs.

Here, we examine the significance of several sequence motifs identified in the *R. leguminosarum* bv. *trifolii rosR* upstream region in the expression of this gene via transcriptional fusions of the *rosR* upstream region with *lacZ.* The level of *rosR* expression and its correlation with EPS production and biofilm formation are assessed in the presence of phosphate and clover root exudates.

## 2. Results and Discussion

### 2.1. Identification of Putative Regulatory Motifs in the rosR Upstream Region

**Figure 1:**
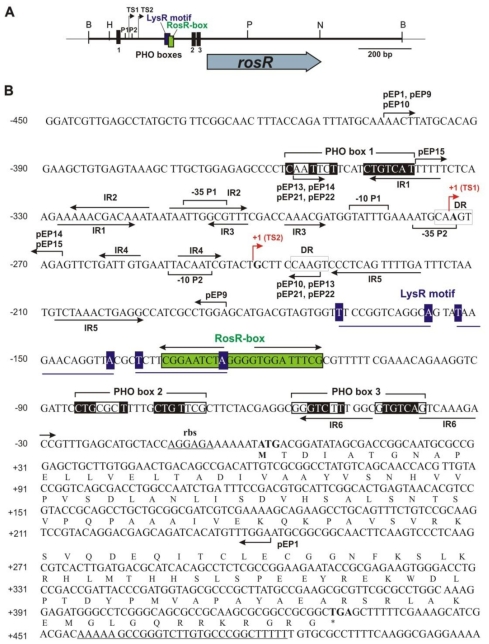
**(A)** Physical and genetic map of plasmid pB31 carrying *R. leguminosarum* bv. *trifolii* 24.2 *rosR* gene. The blue arrow below the map shows the direction of *rosR* transcription. B, *Bam*HI; H, *Hin*dIII; P, *Pst*I; N, *Not*I. P1 and P2 are promoter sequences, and TS1 and TS2 transcription start sites. **(B)** Nucleotide sequence of 960-bp fragment containing *rosR* gene with the upstream region. The amino acid sequence of RosR is given in the single letter code. The −35 and −10 hexamers of P1 and P2 promoters, and PHO boxes are marked by square brackets. Nucleotides identical to the PHO box consensus sequence are shaded in black. TS1 and TS2 are marked by red arrows. RosR-box is shaded in green. LysR motif is underlined and conserved nucleotides are shaded in dark blue. Inverted repeats IR1 to IR6 are marked by inverted arrows, and direct repeats are marked by white boxes. Over lined short arrows indicate the upstream and downstream endpoints of PCR fragments of individual plasmid fusions, respectively. The sequence of ribosome-binding site (rbs) and *rho*-independent terminator are underlined.

Previously, we established that *rosR* expression was driven from two separate promoters: the distal strong P1 promoter and the proximal, weaker, P2, and two *rosR* transcripts of different lengths were identified [36]. These transcripts contained 273-nt and 240-nt long 5′-untranslated regions (5′-UTR), respectively. Such long upstream regions have often been described as target sites for the regulation of gene expression [44,45]. *In silico* sequence analysis of the *rosR* upstream region revealed the presence of many inverted repeats of different lengths (named IR1 to IR6), and among these IR5 was the longest, with 12-bp inverted repeats (Figure 1). The IR2 motif had 11-bp long inverted repeats, and the IR1 and IR6 motifs had 10-bp long inverted repeats. The inverted repeats IR1, IR2, and IR3 were located upstream of the two transcriptional start sites TS1 and TS2. The IR4 motif was located between TS1 and TS2; the IR5 and the IR6 were located below TS2. Direct repeats (DR) and three motifs bearing similarity to the PHO box consensus sequence were also identified in the *rosR* upstream region (Figure 1). PHO boxes located in promoters of phosphate-regulated genes contain two 7-nt direct repeats of the 5′-CTGTCAT-3′ consensus sequence separated by a 4-nt A/T rich spacer, and these motifs are targets for the phosphorylated form of the PhoB regulatory protein [28,46].

Among three potential PHO box-like sequences found in the *rosR* promoter, PHO box 1 located just upstream of the distal P1 promoter comprised a weakly conserved 7-nt direct repeat separated by four nucleotides from a second direct repeat identical to the consensus sequence (Figure 1). Two other PHO boxes were located closer to the translation start codon of *rosR* and among them, PHO box 3 was more conserved than PHO box 2.

**Figure 2:**
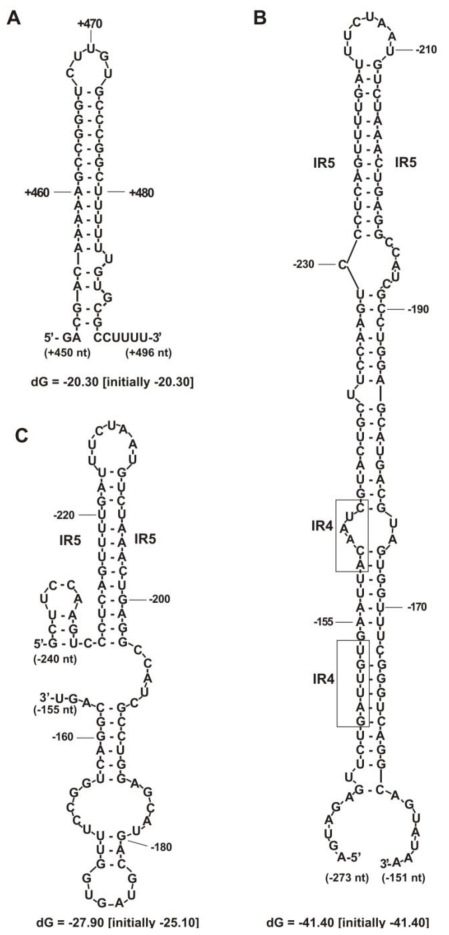
**(A)** The secondary structure of *rho*-independent transcriptional terminator; **(B)** the 5′-upper part of transcript 1 initiating at TS1; and **(C)** transcript 2 initiating at TS2 of *R. leguminosarum* bv. *trifolii* 24.2 *rosR.* The structures and their ΔG were predicted and displayed using the *mfold* 2.3 program. The sequence of IR4 motif was marked by boxes.

Moreover, a LysR motif, which is a putative target site for proteins belonging to the family of LysR transcriptional regulators, was identified just upstream of the RosR-box. Starting from the first transcription start site (position −273 bp), a 770-nt long transcript was generated. RNA synthesis from the TS2 (position −240 bp) resulted in a 737-nt long transcript. Downstream of the TGA stop codon of *rosR*, a palindrome sequence of a *rho*-independent transcription termination site was found (from +452 to +490 bp). This transcriptional terminator was a 39-base long sequence containing two 12-nucleotide inverted repeats separated by 5 nt, which formed a very stable stem structure with an energy of −20.3 kcal/mol (Figure 2A).

Secondary structure analysis of the *rosR* RNA transcripts initiated at TS1 and TS2 revealed the presence of several additional sequences, which stabilized their structures, especially in the upper part of both transcripts. Three stem structures were generated from the first 120 nucleotides of transcript 1 with a total energy of −41.4 kcal/mol, which were substantially stronger than the very stable structure of the transcriptional terminator (Figure 2B). These included inverted repeats IR5, which were located on the top of this structure. This sequence also played a significant role in stabilizing the upper part of the secondary structure of the shorter transcript 2. Within the first 90 nt of the transcript 2, a structure containing two stems was formed with a total energy of −27.9 kcal/mol (Figure 2C).

### 2.2. Functional Analysis of the Putative Regulatory Motif IR4 in the rosR Upstream Region

Previously, we established that deletion of a short DNA fragment in the *rosR* upstream region (from −232 to −268 bp), located just downstream of the P1 promoter, resulted in a very high increase of *rosR* transcription [42]. In this fragment, IR4 inverted repeats have been identified via *in silico* analyses.

**Figure 3:**
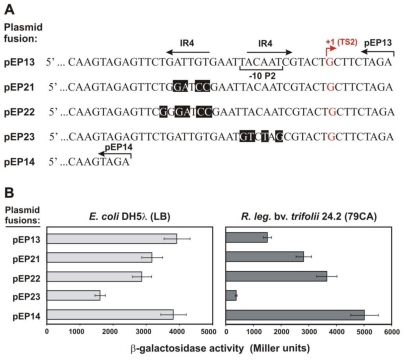
(**A**) The 3′-end nucleotide sequence of selected pEP *rosR-lacZ* fusions. The −10 sequence of P2 promoter is marked by square brackets. IR4 motif is marked by inverted arrows. Transcription start site TS2 is marked by red arrow and the letter G. Over lined arrows indicate the 3′-end of the insert in the individual fusions. Nucleotides changed in the IR4 sequence are shaded in black; (**B**) Transcriptional activity of pEP fusions assayed in *E. coli* DH5α and *R. leguminosarum* bv*. trifolii* 24.2. Data shown are the mean ± SD (n = 4).

To assess the significance of IR4 motif in *rosR* transcription, we used the previously described pEP13 and pEP14 *rosR-lacZ* fusions [42]. pEP14 contained a fragment of the *rosR* upstream region which was 36-bp shorter at the 3′ end than the insert of pEP13. β-galactosidase activity in pEP14 was *ca*. three-fold higher than in pEP13 when studied in Rt24.2. In contrast, *rosR* transcription for both fusions was at the same level in *E. coli* DH5α, suggesting that some element(s) specific for the rhizobial background could be engaged in the repression of *rosR* expression (Figure 3). In the 36-bp fragment, 6-bp-long inverted repeats, named IR4, separated by a 5-nt spacer were identified (Figures 1 and 3). The second part of IR4 partially overlapped the −10 hexamer of the proximal P2 promoter. Site-directed mutagenesis of both parts of IR4 present in pEP13 was performed, and a set of fusions was obtained (pEP21-pEP23) (Figure 3A). β-galactosidase activity was determined for these plasmids and compared with pEP13 and pEP14 control fusions in both *E. coli* and Rt24.2 backgrounds (Figure 3B). In the cases of pEP21 and pEP22, with substitutions in the first part of IR4, *rosR-lacZ* expression in Rt24.2 increased 1.87-fold and 2.44-fold, respectively, in comparison to pEP13, confirming that the IR4 motif was most likely engaged in the negative regulation of *rosR* transcription. In contrast, the mutation of the second part of IR4 overlapping the −10 hexamer of P2 (pEP23 fusion) resulted in a 4-fold decrease of *rosR-lacZ* expression in relation to pEP13 (Figure 3B). These data indicate that the P2–10 hexamer is indispensable for *rosR* expression and positively affects the transcription of this gene.

To elucidate the role of the IR4 motif in the stabilization of RNA, the secondary structure of the *rosR* transcript initiated at TS1 and containing mutated sequences in both parts of the IR4 was analyzed.

Three stem structures generated from the first 120 nucleotides of the wild type transcript 1 had a total energy of −41.4 kcal/mol, whereas the mutation of the first part of IR4 (corresponding to the pEP21 and pEP22 fusions) resulted in a moderate decrease of RNA secondary structure stability (dG = −35.4 and −35.2 kcal/mol, respectively). The mutation of the second part of IR4 slightly affected the *rosR* RNA secondary structure stability (dG = −39.6 kcal/mol). It is plausible that the first part of the IR4 motif functions as a target site for some unidentified repressor protein, and its role in the *rosR* RNA secondary structure stabilization is rather minor. The second part of IR4 that matches the P2–10 hexamer, primarily functions as a binding site of RNA polymerase. Taken together, the results described suggest that the mRNA transcript of this region forming an IR4 loop structure could modulate the level of *rosR* expression.

### 2.3. Functional Analysis of Other Putative Regulatory Motifs in the rosR Upstream Region

Phosphate is one of the most important nutrients for bacteria and is often limited in the environment [47]. *S. meliloti* mutants defective in phosphate transport did not fix nitrogen [48], suggesting a significant role of this nutrient for effective symbiosis. In this bacterium, phosphate regulates a considerable number of genes, among them genes involved in succinoglycan (EPS I) and galactoglucan (EPS II) biosynthesis, through PhoB response regulator forming a two-component regulatory system with PhoR as the sensor kinase [20,21,27,28,44,46]. Whereas phosphate-dependent exopolysaccharide genes regulated by PhoB have been well studied in *S. meliloti* [20,44], the phosphate regulation of genes involved in EPS synthesis in *R. leguminosarum* has not been investigated. Computational analysis revealed the presence of *phoB* (RL0547-position 591, 764–592, 447 nt) in *R. leguminosarum* bv. *viciae* 3841 genome [13], encoding a putative PhoB with 100% amino acid identity to *R. leguminosarum* bv. *trifolii* WSM1325 PhoB, 93% identity to *S. meliloti* PhoB and 54% identity to *E. coli* PhoB [49], indicating a high conservation of the structure and, plausibly, the function of this protein.

**Figure 4:**
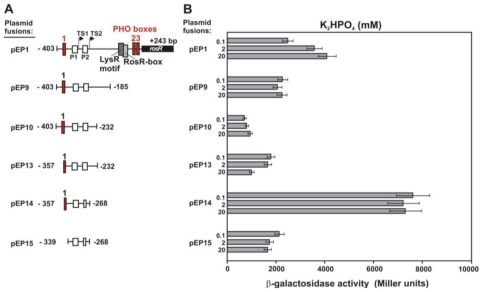
Effect of phosphate on the transcriptional activity of *R. leguminosarum* bv. *trifolii* 24.2 *rosR*. (**A**) Schematic map of pEP *rosR-lacZ* fusions containing different 5′- and 3′-end deletions of the *rosR* upstream region. Promoters P1 and P2 are marked by white boxes, and 5′ part of *rosR* open reading frame is marked by black box. Transcription start sites TS1 and TS2 are marked by angled arrows. The RosR-box, LysR motif and PHO boxes are marked by light gray, dark gray and red rectangles, respectively; (**B**) Effect of phosphate on the transcriptional activity of *rosR* assayed in the *R. leguminosarum* bv. *trifolii* 24.2 strain containing different pEP fusions. For each strain, β-galactosidase activity was assayed in triplicate. Data shown are the mean ± SD.

To establish whether the sequence of PHO boxes identified *in silico* played a role in the regulation of *rosR* expression, *rosR-lacZ* transcriptional fusions were investigated *in vivo. R. leguminosarum* bv. *trifolii* 24.2 wild type (Rt24.2) harboring the pEP1 carrying the longest promoter region (403-bp) or its derivatives containing the promoter region truncated at the 3′- or 5′-end were cultured in the presence of phosphate, and β-galactosidase activities were measured (Figure 4).

In general, *rosR* demonstrated a high level of expression when the longest fusion, pEP1, was examined in standard M1 medium, confirming the presence of strong promoter in its upstream region. Transcription of the pEP1 increased in the presence of increasing concentrations of phosphate, and the highest level (1.68-fold) was observed in the presence of 20 mM K_2_HPO_4_ (Figure 4B). Transcription of pEP1 decreased in the presence of 40 mM K_2_HPO_4_ (data not shown).

In order to establish which of the three PHO boxes identified could be involved in phosphate-dependent regulation of *rosR* transcription, strain Rt24.2 bearing fusions with different deletions was cultured in the individual concentrations of phosphate, and β-galactosidase activities were determined (Figure 4B). The deletion of the 3′-end of the *rosR* upstream region containing the RosR-box, the LysR motif, and two PHO boxes, resulted in a decrease of *rosR-lacZ* expression in the pEP9 fusion in relation to pEP1. This result confirmed the essential role of these regulatory elements in *rosR* expression. The transcription in the pEP10 fusion that had a progressively enlarged deletion of the 3′-end was further diminished. The deleted fragment contained IR5, indicating that this motif, predicted to play a dominant role in RNA secondary structure stabilization, is likely responsible for stimulation of *rosR* expression. Simultaneous deletions of 46 bp in the 5′-end of the *rosR* untranscribed region and 36 bp in the 3′-end in pEP14 resulted in a nearly 4-fold increase of the expression of this gene (Figure 4B). In contrast to pEP13, pEP14 lacked IR4 motif which played the substantial role in the negative regulation of *rosR* transcription.

Unlike the longest pEP1 fusion, the transcriptional activities of the remaining pEP9–pEP15 fusions seem to be phosphate independent (Figure 4B). pEP14, containing a putative PHO box 1 lacking the first nucleotide at the 5′-end, showed a very high transcriptional activity which was rather independent of (or only weakly dependent on) phosphate concentration. This finding excludes the role of PHO box 1 in *rosR* expression, despite its relatively high identity to the PHO box consensus sequence. The pEP15 fusion with a deletion of the full-length PHO box 1 demonstrated a very low level of *rosR* expression, which was totally independent of phosphate. These data show that PHO boxes 2 and 3, located close to the translation start site, may be engaged in phosphate-dependent regulation of *rosR* transcription.

**Figure 5:**
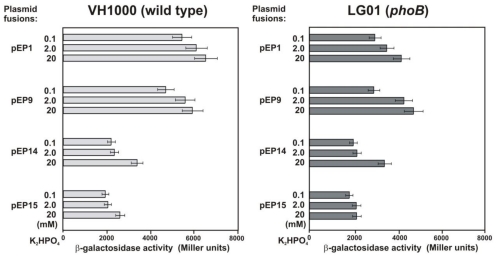
Effect of phosphate on the transcriptional activity of *rosR* as assayed in the *E. coli* VH1000 wild type strain and *phoB* mutant containing different *rosR-lacZ* fusions. For each strain, β-galactosidase activity was assayed in triplicate. Data shown are the mean ± SD.

To further assess the function of the PHO boxes identified in the *rosR* upstream region, the following plasmids were introduced into *E. coli* wild type and *phoB* mutant: pEP1 containing all three PHO boxes, pEP9 containing exclusively PHO box 1, pEP14 containing PHO box 1 without the first nucleotide, and pEP15 lacking the PHO box 1, and β-galactosidase activities were measured (Figure 5).

In general, *rosR* expression in the pEP1 was significantly higher in the wild type than in the *phoB* mutant. In a low-phosphate medium (0.1 mM), the transcription in pEP9 was lower in relation to pEP1 in the wild type strain (P < 0.05, Student’s test), but this difference disappeared in the *phoB* mutant. For pEP14 and pEP15, similar profiles of *rosR* expression were found in both genetic backgrounds. Moreover, a significant decrease of *rosR-lacZ* expression was observed for pEP14 in comparison to pEP1. The pEP15 fusion, lacking all three PHO box-like sequences, showed a very low *rosR* expression independent of phosphate in the wild type and the *phoB* mutant (Figure 5). These results indicate that PhoB from *E. coli* stimulates *rosR* transcription, most likely by binding to PHO boxes 2 and/or 3.

All above data suggest that PHO boxes 2 and/or 3, rather than PHO box 1, might play a role in phosphate-dependent regulation of *rosR* expression, although this effect is not considerable. In general, PhoB protein functions as a positive regulator, which induces the expression of genes belonging to Pho-regulon under phosphate limitation. However, Bardin *et al.* [50] demonstrated that the expression of *orfA-pit* encoding a phosphate transport membrane protein in *S. meliloti* is repressed upon P_i_ starvation. This finding is in contrast to *E. coli,* where *pit* genes are constitutively expressed [51]. In *S. meliloti*, the majority of PhoB-regulated genes possess PHO boxes located upstream of their promoters [28], whereas for *orfA-pit* and *fixN3* this motif is located just upstream of their putative translation start [50], similarly to PHO boxes 2 and 3 in the *rosR* promoter.

### 2.4. Functional Analysis of a Putative LysR Motif

A well-conserved LysR motif (T-N_11_-A)_3_, possibly interacting with proteins of the LysR family, was identified in close vicinity of the RosR-box and two PHO boxes in the *rosR* upstream region (Figure 1). One of these LysR proteins is NodD involved in the transcription activation of *nod* genes responsible for Nod factor synthesis in the presence of flavonoids. To assess whether NodD influences *rosR* expression, fusions pEP1 containing the LysR motif and pEP9 lacking it, were introduced into *R. leguminosarum* bv. *trifolii* ANU843 wild type and ANU851 carrying a mutation in *nodD*, and β-galactosidase activity was examined in the presence and absence of clover root exudates (Table 1).

For ANU843(pEP1) growing in the presence of 10 μM exudates, a moderate increase of β-galactosidase activity (1.37-fold) was found. In the ANU851(pEP1) *nodD* mutant, the effect of exudates was negligible (1.04-fold increase). In the case of pEP9, lacking the LysR motif, *rosR* expression was similar in the wild type and the *nodD* mutant, and almost negligently responsive to exudates. These data suggest that the LysR motif present in the *rosR* upstream region might be functional, recognized by NodD and may influence *rosR* transcription.

The LysR motif was described as a binding site for several transcription factors containing a helix-turn-helix domain, among them NodD [52]. In *S. meliloti*, SyrM, also belonging to LysR-type transcription factors, is involved in the activation of the EPS I synthesis genes [30,31], but we did not find a homologous protein encoded by *R. leguminosarum* genome.

### 2.5. Effect of Phosphate and Clover Root Exudates on EPS Production

**Figure 6:**
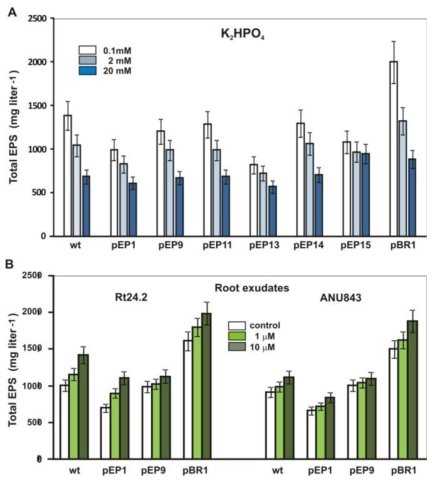
Effect of phosphate and clover root exudates on EPS production in *R. leguminosarum* bv. *trifolii*. **(A)** EPS production in the presence of different concentrations of phosphate was assayed in the Rt24.2 wild type and its derivatives bearing different pEP fusions and pBR1 with additional *rosR* copies; **(B)** EPS production in the presence of clover root exudates assayed in Rt24.2 and ANU843 wild type strains and their derivatives. The given values are the mean ± SD of triplicate assays.

In our previous study, it was found that EPS synthesis in *R. leguminosarum* was positively controlled by RosR binding to the RosR-box in the promoter region of *rosR* and *pssA* encoding the first IP-glycosyl transferase [36]. Here, the effect of phosphate and root exudates on EPS production was studied in Rt24.2 wild type, Rt24.2(pEP1) carrying the full-length *rosR* upstream region on a low copy plasmid pMP220, Rt24.2 carrying the pEP9-pEP15 fusions, and Rt24.2(pBR1) harboring additional copies of *rosR* on the pBBR1MCS-2 plasmid (Figure 6).

Among tested growth conditions, the most effective production of this polysaccharide was observed in M1 with 0.1 mM phosphate, and the wild type strain produced 1.94-fold more EPS under phosphate limitation (0.1 mM) than in the presence of high phosphate concentration (20 mM). The stimulation of EPS synthesis in the phosphate starvation was even more visible for Rt24.2 (pBR1) bearing additional *rosR* copies*,* confirming positive regulation of EPS synthesis by RosR (1.42-fold more EPS than in the wild type) (Figure 6A). EPS production in all of the tested strains was reduced with increasing concentrations of phosphate, showing a negative effect of this compound even though the *rosR* transcription (pEP1 fusion) was stimulated by up to 20 mM phosphate. Introduction of multiple copies of the *rosR* upstream region containing the RosR-box, being the target site for RosR binding, caused a significant reduction of the amount of EPS (pEP1 fusion). Because in the case of pEP14, which almost totally lacked transcriptional responsiveness to phosphate, the strain carrying this fusion still produced more EPS under phosphate limitation than phosphate-rich condition, these results suggest that other chromosomal genes, besides *rosR*, involved in EPS production might also be regulated by phosphate. This demonstrates complexity of phosphate-dependent regulation of EPS synthesis in *R. leguminosarum*. The EPS synthesis in this bacterial species seems to be much more sensitive to phosphate concentration than EPS I production in *S. meliloti*, being efficiently synthesized under very high concentration of phosphate (above 150 mM). The expression of *S. meliloti exoYFQ* operon engaged in EPS I synthesis was affected by phosphate concentration and was PhoB-dependent, although PHO box-like sequence identified in its promoter had a relatively low sequence identity with the PHO box consensus [44]. In low-phosphate medium, galactoglucan was efficiently produced [25], and both WggR and PhoB regulatory proteins were required for a maximal induction of the transcription of the EPS II biosynthesis gene cluster under P_i_ starvation [20].

In the case of clover root exudates, a moderate stimulation of EPS synthesis was found for Rt24.2 (1.43-fold) and ANU843 (1.28-fold) wild type strains cultured in M1 supplemented with 10 μM exudates, and full congruence was observed between *rosR* transcription and EPS production (Table 1 and Figure 6B). This indicated that root exudates positively affected EPS production in *R. leguminosarum* via stimulation of *rosR* transcription in the presence of the LysR motif.

### 2.6. Biofilm Formation by R. leguminosarum bv. trifolii on Abiotic Surfaces in the Presence of Phosphate and Clover Root Exudates

**Figure 7:**
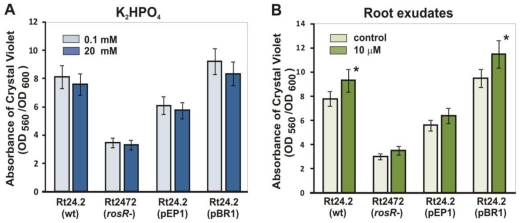
(**A**) Effect of phosphate and (**B**) clover root exudates on biofilm formation by *R. leguminosarum* bv. *trifolii* 24.2 wild type and its derivatives. For each strain, the assays were performed in triplicate and data shown are the means ± SD. * indicates statistically significant differences.

Rhizobia, like many other bacteria, form surface-attached communities, known as biofilm, which most likely protect the bacterium against harmful environmental factors or nutrients deficiency [1,2,53]. Biofilm formation in the presence of phosphate and flavonoids was studied using Rt24.2 wild type, the Rt2472 *rosR* mutant, Rt24.2 harboring pEP1 with additional copies of the *rosR* upstream region, and Rt24.2 carrying pBR1 with multiple *rosR* copies (Figure 7).

Strain Rt24.2(pEP1) formed about 70% of control biofilm, and the Rt2472 *rosR* mutant formed about 3-fold less biofilm than Rt24.2 wild type in the presence of 20 mM phosphate, which most likely reflected the decrease of EPS synthesis under these conditions (Figure 7A). Phosphate limitation (0.1 mM) caused a slightly increase of the amounts of biofilm formed by Rt24.2 and its derivative Rt24.2(pBR1), but the differences observed between the low-phosphate and the high-phosphate conditions were not statistically significant.

On the other hand, the amounts of biofilm formed by Rt24.2 harboring extra copies of *rosR* were higher than those produced by the control strain, especially under low-phosphate. This was in accordance with the earlier observed increase of EPS production under these conditions, confirming the positive role of EPS in biofilm formation (Figure 6A and Figure 7A).

Root exudates exerted a positive effect on biofilm formation by the strains used (Figure 7B). This result was in agreement with the higher level of *rosR* expression and EPS synthesis in the presence of flavonoid extract (Table 1 and Figure 6B).

Recent studies of rhizobial species showed that they form biofilms either on abiotic surfaces and on host plant roots [14–16,53]. From among several factors influencing biofilm formation in rhizobia, the ability to produce EPS, the degree of EPS polymerization, and functional quorum-sensing systems are the most important [1,14,16,17]. EPS non-producing *pssA* mutant of *R. leguminosarum* bv. *viciae* was found to be defective in attachment and biofilm formation both *in vitro* and on root hairs [15,16], confirming the crucial role of EPS in biofilm formation. Also, the *R. leguminosarum* bv. *trifolii pssA* mutant previously described by us [41], which does not produce EPS, formed significantly less (18.9%) biofilm than the wild type strain on abiotic surfaces. Moreover, both *rosR* and *pssA* mutants of the Rt24.2 strain demonstrate a decreased ability to attach to clover roots (the number of bacteria attached to host plant roots after 48-h incubation was about 7-fold [43] and 20-fold lower in the case of the *rosR* and the *pssA* mutants, respectively, in comparison to the wild type strain).

In *S. meliloti,* a key role of EPS II in mature biofilm formation for the correct interaction with the host plant has been shown [17]. Also*,* common *nodD1ABC* genes, whose products synthesize core Nod factor, were required for the establishment of a mature biofilm [54]. Moreover, some nutritional and environmental conditions, such as increasing concentrations of sucrose, phosphate and calcium enhance biofilm formation, whereas pH and extreme temperature negatively affect this process in *S. meliloti* [55].

In this work, we found that phosphate starvation and the presence of clover root exudates slightly increased biofilm development by *R. leguminosarum* bv. *trifolii*. Similarly, phosphate limitation only slightly enhanced biofilm formation by the plant pathogen *Agrobacterium tumefaciens* through the PhoR-PhoB regulatory system [56].

### 2.7. Effect of Phosphate and Flavonoids on Symbiosis of Rt24.2 and Its Derivatives with Clover

Previously, we established that the symbiotic efficiency of the *rosR* mutant was significantly decreased [41]. This mutant induced 2-fold fewer nodules on clover roots than the wild type strain, which were occupied by significantly lower numbers of bacteria and, as a consequence, a low shoot mass of the infected clover plants was observed. The *pssA* mutant, which did not produce EPS, induced a 3-fold lower number of nodules than the wild type, and those nodules were hardly occupied by bacteria. This resulted in a significant decrease in the wet shoot mass (60%) of clover plants in comparison to plants inoculated with the wild type strain, confirming the significant role of EPS in host plant infection and effective symbiosis. On the other hand, we observed that multiple copies of *rosR* and *pssA* genes significantly enhanced EPS production, competitiveness and clover nodulation in *R. leguminosarum* bv. *trifolii* [41].

**Figure 8:**
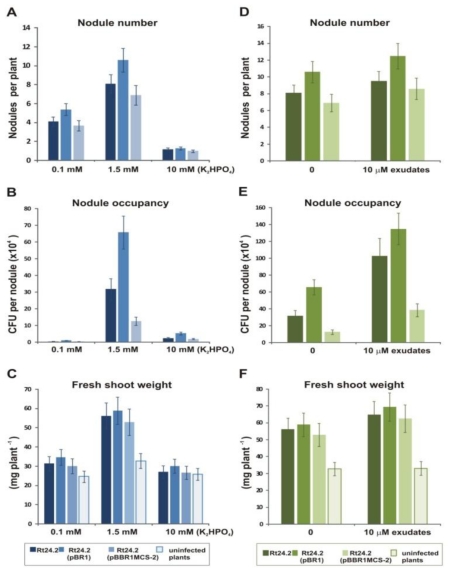
Effect of phosphate **(A–C)** and root exudates **(D–F)** on symbiosis of *R. leguminosarum* bv. *trifolii* 24.2 and its derivatives Rt24.2(pBR1) and Rt24.2(pBBR1MCS-2) with clover plants. The tested symbiotic parameters are: the nodule number (**A** and **D**), nodule occupancy (**B** and **E**) and fresh shoot weight (**C** and **F**). Plants were harvested 28 days after inoculation. Given values are averages of three independent experiments with 20 plants for each treatment. For nodule occupancy, values are means ± SD of 12 nodules.

In this work, the effect of phosphate and flavonoids on nodulation, nodule occupancy, and plant growth was examined using Rt24.2 wild type and its derivatives Rt24.2(pBR1) carrying additional *rosR* copies and Rt24.2 harboring the pBBR1MCS-2 vector (Figure 8). Nitrogen-free medium containing 1.5 mM phosphate was found to be optimal for symbiotic performance. Under phosphate-rich conditions (10 mM), a considerable reduction in nodule numbers, nodule occupancy, and fresh shoot weights was noticed (Figure 8A–C). This negative effect was even more drastic than the effect of phosphate limitation (0.1 mM), with the exception of nodule occupancy (Figure 8B).

To assess the effect of exudates on symbiosis of Rt24.2 and its derivatives, bacteria were treated for 3 h with flavonoids before plant inoculation (Figure 8D–F). This pretreatment of rhizobia resulted in a faster induction of a higher number of nodules, significantly improved nodule occupancy and higher masses of shoots. These results showed that root exudates from a compatible legume host helped the microsymbionts in the first steps of symbiosis, probably by enhancing the synthesis of Nod factors and, possibly, EPS.

## 3. Experimental Section

### 3.1. Bacterial Strains, Plasmids, and Growth Conditions

The bacterial strains, plasmids, and oligonucleotide primers used in this study are listed in Table 2.

*R. leguminosarum* bv. *trifolii* strains were grown in 79CA medium with 1% glycerol as a carbon source [62], in tryptone-yeast (TY) medium, and in M1 minimal medium [60] containing 1% glycerol and 2 mL L^−1^ vitamin stock solution [63] at 28 °C. *E. coli* strains were grown in Luria-Bertani (LB) medium at 37 °C [60]. To study the effect of phosphate on *rosR* expression, EPS production, and biofilm formation*,* the strains were cultured in M1 medium containing 20 mM morpholinopropane sulfonate (MOPS) supplemented with appropriate concentrations of K_2_HPO_4_. To establish the influence of clover root exudates on the expression of *rosR-lacZ* fusions, EPS and biofilm formation, the strains were grown in 5 mL M1 medium supplemented with vitamins and 10 μM clover root exudates. When required, antibiotics for *E. coli* and *R. leguminosarum* were used at the following final concentrations: kanamycin, 40 μg mL^−1^; ampicillin, 100 μg mL^−1^; tetracycline, 10 μg mL^−1^; and nalidixic acid, 40 μg mL^−1^.

### 3.2. DNA Methods and Sequence Analysis

Standard techniques were used for plasmid isolation, restriction enzyme digestion, agarose gel electrophoresis, cloning, and transformation [60]. For PCR amplification, plasmid or genomic DNAs isolated from *R. leguminosarum* bv. *trifolii* as templates and *Pfu* DNA polymerase (Promega, Madison, Wl, USA) or *Taq* DNA polymerase (Sigma-Aldrich, St. Louis, MO, USA) were used. The primers used in this study are listed in Table 2. Sequencing was performed using the BigDye terminator cycle sequencing kit (Applied Biosystems, Foster City, CA, USA) and the ABI Prism 310 DNA sequencer. Database searches were done with the BLAST and FASTA programs available at the National Center for Biotechnology Information (Bethesda, MD, USA) and the European Bioinformatic Institute (Hinxton, UK). The searches for PHO box motifs and inverted repeats (IR) in the promoter region of *R. leguminosarum* bv. *trifolii rosR* were performed with the “Malign” [64] and “Fuzznuc” [65] programs. RNA secondary structures were predicted using the RNA folding software *mfold* version 3.2 [66] using the default settings.

### 3.3. Construction of Transcriptional rosR-lacZ Fusions with Mutated IR4 Sites

To construct plasmid fusions containing specific *rosR* promoter fragments, the broad-host-range plasmid pMP220 carrying a promoterless *lacZ* gene was used. A set of promoter deletions was generated on the basis of the 1.2-kb insert of pB31 bearing the entire *rosR* promoter. For construction of the pEP21 fusion, two primer pairs, pROS2/pREW2 and pRBAM1/pREW3, were used (Table 2), giving amplicons of 116-bp and 67-bp, respectively. The 116-bp fragment was digested with *Eco*RI and *Bam*HI enzymes, and the 67-bp amplicon with *Bam*HI and *Xba*I enzymes. Both the *Eco*RI-*Bam*HI and the *Bam*HI-*Xba*I fragments were ligated and cloned into the *Eco*RI and *Xba*I sites of the pUC19 vector, giving construct pMJ221. For pEP22 fusion, the same approach was used as for pEP21, except that different primer pairs, pROS2/pREW4 and pRBAM1/pREW3, were used. The obtained amplicons of 116-bp and 67-bp were digested with *Eco*RI/*Bam*HI and *Bam*HI/*Xba*I, respectively, ligated, and cloned into the *Eco*RI and *Xba*I sites of the pUC19, giving plasmid pMJ222. The inserts of both pMJ221 and pMJ222 were verified by sequencing. For pEP23 fusion, the primers pROS2 and pREW1 were applied. The 139-bp PCR fragment was digested with *Eco*RI and *Xba*I and cloned into the respective sites of the pUC19 vector. The resulting plasmid was named pMJ223, and its insert was verified by sequencing. Then, the *Eco*RI-*Xba*I fragments of pUC19 derivatives were recloned into the respective sites of pMP220, resulting in plasmids pEP21, pEP22, and pEP23 (Table 2). The final constructs were introduced into *E. coli* S17-1 by transformation, and then transferred from *E. coli* S17-1 into *R. leguminosarum* bv. *trifolii* 24.2 (Rt24.2) via biparental conjugation.

### 3.4. β-Galactosidase Assay

Rt24.2 derivatives containing *rosR-lacZ* fusions were grown for 24 h in 79CA or M1 medium supplemented with tetracycline. *E. coli* VH1000 wild type and *phoB* mutant strains bearing pEP fusions were cultured for 24 h in M1 medium supplemented with tetracycline and different concentrations of phosphate. The assay for β-galactosidase activity was carried out as described previously [42].

### 3.5. EPS Isolation

For EPS isolation, 5-mL cultures of *R. leguminosarum* bv. *trifolii* strains were grown in M1 medium supplemented with 1% glycerol and Dilworth’s vitamins for 3 days at 28 °C in a rotary shaker. To study the effect of the different factors on the level of EPS produced by *R. leguminosarum* bv. *trifolii*, the strains were cultured in M1 medium supplemented with 10 μM root exudates, 1% glycerol as a carbon source, and appropriate concentrations of K_2_HPO_4_. EPS was precipitated from culture supernatants with 4 volumes of cold 96% ethanol, and then collected by centrifugation, lyophilized, resolved in water and analyzed for carbohydrates according to Loewus [67]. Total sugar content was calculated as glucose equivalents.

### 3.6. Biofilm Formation Assay

The biofilm formation assay was done in microtiter-plates as described by Rinaudi and Gonzalez [17]. Briefly, *R. leguminosarum* bv. *trifolii* strains were grown in M1 medium supplemented with Dilworth’s vitamins and different concentrations of the examined compounds at 28 °C for 48 h. The cultures were diluted to an OD_600_ of 0.3, introduced in 100 μL aliquots into microplate wells, and incubated at 28 °C for 48 h without shaking. Then, bacterial growth was quantified by measuring the OD_600_. The liquid was removed, and each well was washed three times with 150 μL of 0.85% NaCl, stained for 15 min with 150 μL of 0.1% crystal violet, and rinsed three times with 150 μL of water. After drying, biofilm formation was quantified by the addition of 150 μL of 95% ethanol and measurement of OD_560_ using a Benchmark Plus™ microplate reader (Bio-Rad Laboratories, Hercules, CA, USA).

### 3.7. Preparation of Exudates from Sprouted Seeds of Clover

Clover seeds after surface sterilization (0.1% HgCl_2_, 3 min, 70% ethanol, 3 min) were shaken in 1 L sterile water (proportion: 1 mL of water per 2 seeds) in darkness for 4 days at 28 °C [43]. Then, the supernatant was two times extracted with ethyl acetate in a 10:1 (volume/volume) ratio. Ethyl acetate was drained, and the pellet was solubilized in 10 mL 96% ethanol and stored at 4 °C. To determine the amount of substances present in the ethanol fraction of clover root exudates, 0.5 mL of this fraction was dried and weighed, and the obtained mass was calculated using the molecular mass of 7,4′-dihydroxyflavone as a standard flavonoid.

### 3.8. Plant Tests

Red clover (*Trifolium pratense* cv. Diana) seeds were surface sterilized, germinated, and grown on Fåhraeus medium slants [60]. 5-day-old seedlings were inoculated with bacterial suspensions of an OD_600_ of 0.2 (200 μL per plant) and grown under natural light supplemented with artificial light (14 h-day at 24 °C and 10 h-night at 18 °C) in a greenhouse. After 4 weeks, the number of nodules formed on clover roots was counted, and wet mass of clover shoots was estimated. For nodule occupancy, 4-week-old nodules were surface-sterilized, and each nodule was crushed in 100 μL of 79CA medium containing 0.3 M glucose; the obtained suspensions were plated in dilutions on plates containing 79CA agar.

## 4. Conclusions

Our previous studies indicated that RosR is an essential regulatory protein involved in EPS synthesis in *R. leguminosarum* bv. *trifolii* [36,42,43]. Mutation of *rosR* resulted in a 3-fold decrease of EPS production, whereas additional *rosR* copies caused a *ca.* 2-fold increase of EPS, confirming the significant role of RosR as a positive regulator of EPS production.

In this work, we found that the expression of *rosR* is very complex and is modulated by phosphate and clover root exudates. In the long *rosR* upstream region, besides the RosR-box that binds RosR and autoregulates *rosR* expression, several regulatory motifs were identified which showed a significant identity to consensus sequences recognized by PhoB and LysR-type proteins associated with phosphate- and flavonoid-dependent gene regulation. The complexity of *rosR* regulation is additionally enhanced by at least six inverted repeats (IR1–IR6) found in this region. The transcripts initiated from the distal P1 and proximal P2 promoters could form very stable stem-loop secondary structures, which could be involved in post-transcriptional regulation, affecting the stability of *rosR* transcripts. mRNA degradation is an important mechanism for controlling gene expression in bacteria and the lifetimes of the individual transcripts can differ significantly depending on various environmental conditions [68,69]. The *rosR* transcripts initiated at TS1 and TS2 contain a long 5′-untranslated region (5′-UTR) (273-nt and 240-nt, respectively) that might be potentially engaged in the regulation of transcription and translation initiation in response to specific proteins or signals [70,71].

In this study, we found that a deletion of a 36-bp fragment located downstream of TS1 and containing 6-nt inverted repeats IR4 caused a three-fold increase of *rosR* expression driven from the P1 promoter (pEP14 in relation to pEP13). Site-directed mutagenesis of the first part of the IR4 motif confirmed that this sequence negatively regulates *rosR* transcription in Rt24.2, and the second part of the IR4 overlapping the P2–10 sequence plays a significant role in the activation of this process, primarily by binding of RNA polymerase. Because introduced mutations did not significantly affect the stability of RNA secondary structures, we concluded that the IR4 motif could be a potential target for some as yet unidentified rhizobial *trans*-acting factor, which negatively regulates *rosR* expression.

Here, we studied the influence of phosphate and root exudates on *rosR* expression and its link with EPS production and biofilm formation. Our data indicate that EPS synthesis and biofilm formation in *R. leguminosarum* bv. *trifolii* was stimulated in the presence of clover root exudates and under phosphate limitation. Root exudates positively affected EPS production, which was in concordance with a moderate increase of *rosR* transcription in the presence of flavonoids and the LysR motif in pEP1. In the case of phosphate, a negative effect of this compound on EPS production was observed. The EPS synthesis in *R. leguminosarum* seems to be much more sensitive to phosphate concentration than EPS I production in *S. meliloti*, being efficiently synthesized under very high concentration of phosphate (above 150 mM).

Taken together, the presented data indicate that at least four proteins (RosR, PhoB, NodD and the repressor binding the IR4) influence *rosR* transcription by binding to specific DNA consensus sites and, consequently, modulate EPS production in the presence of suitable external nutritional signals. These results are in agreement with published data demonstrating that the activity of most bacterial promoters depends on multiple nutritional signals and is controlled by two or more transcriptional factors with each factor relaying one environmental signal [72].

## Figures and Tables

**Table 1:** Effect of clover root exudates and *nodD* mutation on the *rosR-lacZ* transcription in *R. leguminosarum* bv. *trifolii*.

Plasmid fusion	LysR motif	ANU843 (wt)		ANU851 (*nodD*^−^)	

		Clover root exudates (μM)			
		0	10	0	10
pEP1	+	2630 ± 288	3613 ± 352	2682 ± 253	2789 ± 245
pEP9	−	1778 ± 185	1866 ± 191	1709 ± 167	1811 ± 177

**Table 2:** Bacterial strains, plasmids and oligonucleotide primers used in this study.

Strain or plasmid	Relevant characteristics	Reference
***Rhizobium leguminosarum*bv*. trifolii***		
Rt24.2	Wild type, Rif^r^, Nx^r^	[36]
Rt2472	Rt24.2 derivative carrying mini-Tn*5* between 151–152 bp position of *rosR*, Rif^r^, Km^r^	[41]
ANU843	Wild type, Rif^r^	[57]
ANU851	ANU843 derivative carrying Tn5::*nodD*, Km^r^	[57]
***E. coli***		
VH1000	MG1655 derivative, *lacZ*, *lacI*, *pyrE*^+^	[58]
LG01	MG1655 derivative, *phoB519::Tn5*, Km^r^	[59]
**Plasmids**		
pUC19	Cloning and sequencing vector, Ap^r^	[60]
pMP220	IncP, *mob*, promoterless *lacZ*, Tc^r^	[61]
pMJ221	pUC19 containing 126-bp *Eco*RI-*Xba*I fragment of the *rosR* upstream region (based on primers: pROS2/pREW2 and pRBAM1/pREW3)	This work
pMJ222	pUC19 containing 126-bp *Eco*RI-*Xba*I fragment of the *rosR* upstream region (based on primers: pROS2/pREW4 and pRBAM1/pREW3)	This work
pMJ223	pUC19 containing 126-bp *Eco*RI-*Xba*I fragment of the *rosR* upstream region (based on primers: pROS2/pREW1)	This work
pBR1	pBBR1MCS-2 containing 1100-bp *Eco*RI-*Bam*HI fragment with *rosR* of Rt24.2	[41]
pEP1	pMP220 carrying the −403 to +243 bp fragment of the *rosR* coding region	[36]
pEP9	pMP220 carrying the −403 to −185 bp fragment of the *rosR* upstream region	[36]
pEP10	pMP220 carrying the −403 to −232 bp fragment of the *rosR* upstream region	[36]
pEP13	pMP220 carrying the −357 to −232 bp fragment of the *rosR* upstream region	[42]
pEP14	pMP220 carrying the −357 to −268 bp fragment of the *rosR* upstream region	[42]
pEP15	pMP220 carrying the −339 to −268 bp fragment of the *rosR* upstream region	[42]
pEP21	pMP220 containing 126-bp *Eco*RI-*Xba*I fragment from pMJ221	This work
pEP22	pMP220 containing 126-bp *Eco*RI-*Xba*I fragment from pMJ222	This work
pEP23	pMP220 containing 126-bp *Eco*RI-*Xba*I fragment from pMJ223	This work
**Oligonucleotide primers**	**Sequence (5**′→**3**′)^*^	
pROS2	GAGCCCCTGAATTCTTCATCTGTCA	This work
pREW1	AGGGATCTAGAAGCAGTACGCTAGACATTCAC	This work
pREW2	GTAATTCGGATCCAGAACTCTACTTGCA	This work
pREW3	GAAATCAAAACTGAGGGATCTAGAAGCA	This work
pREW4	GTAATTCGGATCCCGAACTCTACTTGCA	This work
pRBAM1	AAATGCAAGTAGAGTTCTGGATCCGAATTA	This work

* Sequences for *Eco*RI, *Bam*HI and *Xba*I restriction sites are underlined.
